# A new species of *Eimeria* Schneider, 1875 from the Serra dos Órgãos National Park, Rio de Janeiro, Brazil, with notes on its endogenous development in the montane grass mouse, *Akodon montensis* Thomas, 1913 (Rodentia: Sigmodontinae)

**DOI:** 10.1007/s00436-017-5707-4

**Published:** 2017-12-11

**Authors:** Marcos Tobias de Santana Miglionico, Lúcio André Viana, Helene Santos Barbosa, Ester Maria Mota, Sócrates Fraga da Costa Neto, Edwards Frazão-Teixeira, Paulo Sergio D’Andrea

**Affiliations:** 1 Programa de Pós-Graduação em Biodiversidade e Saúde, Instituto Oswaldo Cruz/Fiocruz, Rio de Janeiro, RJ 21040-360 Brazil; 2 Departamento de Ciências Biológicas e da Saúde, Universidade Federal do Amapá, Macapá, AP 68903-419 Brazil; 3 Laboratório de Biologia Estrutural, Instituto Oswaldo Cruz/Fiocruz, Rio de Janeiro, RJ 21040-360 Brazil; 4 Laboratório de Patologia, Instituto Oswaldo Cruz/Fiocruz, Rio de Janeiro, RJ 21040-360 Brazil; 5 Laboratório de Biologia e Parasitologia de Mamíferos Silvestres Reservatórios, Instituto Oswaldo Cruz/Fiocruz, Rio de Janeiro, RJ 21040-360 Brazil

**Keywords:** Rodents, Coccidia, *Akodon montensis*, Atlantic Forest, Histopathology, Cricetidae

## Abstract

A total of 53 specimens of the montane grass mouse, *Akodon montensis* Thomas, 1913 were collected in the Serra dos Órgãos National Park (SONP) in November 2014 and July 2015. The fecal material was analyzed, and a prevalence of 7.5% was recorded for a new coccidian species of the genus *Eimeria* Schneider, 1875, with part of its endogenous development recorded in the small intestine. The oocysts of a new coccidian species of genus *Eimeria* are ellipsoidal to subspherical. The wall is bi-layered, c. 1.5 μm (1.3–1.6 μm) thick, outer layer rough. Oocyst (*n* = 126) mean length is 25.3 μm (21.0–28.0 μm), with a width of 20.2 μm (17.0–22.0 μm) and mean length/width (L:W) ratio of 1.3 (1.2–1.4). Polar granule is present, with the oocyst residuum as a large spherical to subspherical globule. Sporocyst shape (*n* = 126) is ellipsoidal, with a mean length of 11.8 μm (9.3–14.4 μm), width of 7.9 μm (6.7–9.3 μm), and mean L:W ratio of 1.5 (1.4–1.7). Sporocysts with nipple-like Stieda body and sub-Stieda body are absent. A sporocyst residuum formed by several globules, usually along the sporocyst wall. This is the first record of *Eimeria* in the montane grass mouse from Brazil.

## Introduction

*Eimeria* spp. are protozoan parasites with worldwide distribution that might cause enteritis in many vertebrate species (Duszynski and Upton 2001). In Brazil, only three species are known to infect Sigmodontinae rodents: *Eimeria oryzomysi* Carini, 1937 from specimens of Oryzomyini rodents; *Eimeria zygodontomyis* Lainson and Shaw, 1990 in *Zygodontomys lasiurus* [= *Necromys lasiurus* (Lund, 1840)], and *Besnoitia akodoni* Dubey, Sreekumar, Rosenthal, Lindsay, Grisard and Vitor, 2003 in *Akodon montensis*.

The rodent family Cricetidae has 130 genera encompassing 681 species, 55 of which are endemic in Brazil. The cricetid genus *Akodon* Meyen, 1833 has 44 species distributed throughout South America (Musser and Carlenton 2005), with 10 species being found in Brazil, on the east coast between Paraíba and Rio Grande do Sul, and inland in highland areas of Minas Gerais, and in the southwest of the state of Mato Grosso do Sul (Bonvicino et al. 2008). The ecology and taxonomy of some common *Akodon* species are well known. They are found in a range of different environments, both pristine and impacted, in addition to their epidemiological importance, as reservoirs of zoonoses (Oliveira et al. 2012; Müller et al. 2013; Teixeira et al. 2014). Four species are found in the Brazilian state of Rio de Janeiro—*Akodon cursor* (Winger, 1887), *Akodon serrensis* (Thomas, 1902), *Akodon paranaensis* Christoff et al., 2000, and *Akodon montensis* Thomas, 1913 (Paglia et al. 2012). These rodents are terrestrial insectivore-omnivores (Graipel et al. 2003).

The present study describes a new species of *Eimeria*, with notes on its endogenous development in the montane grass mouse, *Akodon montensis*.

## Material and methods

### Study area

The study area encompasses a continuous tract of well-preserved Brazilian Atlantic Forest, isolated from human settlements or urban zones. The rodents were captured in the Serra dos Órgãos National Park (SONP), in the municipality of Petrópolis, Rio de Janeiro State, Brazil (22° 27′ 49″ S, 43° 05′ 14.09″ W). This park is located near the center of the Serra do Mar Ecological Corridor (Aguiar et al. 2005) and is one of the principal remnants of Atlantic Forest in the state of Rio de Janeiro.

### Capture of the rodents

Rodents were captured in November 2014 and July 2015 along ten transects in each expedition. The specimens were captured in Tomahawk^®^ (16 × 5 × 5 in.) and Sherman^®^ traps set on the ground, a total of 90 live traps set per day. Additionally, 20 pitfall traps, made of 100-l buckets, were buried in the ground, along four transects, a capture effort of 80 pitfalls traps set per day. Each trapping session lasted for ten consecutive days. All animals were captured alive and euthanized a posteriori for sample collection. Voucher specimens were deposited in the scientific collection of the National Museum (Museu Nacional) at the Universidade Federal do Rio de Janeiro (UFRJ). All the samples were collected during the Rede Bioma (Biome Network) project “Inventories: Patterns of Diversity, Biogeography, and Endemism of Mammals, Birds, Amphibians, Fruit Flies, and Parasites in the Atlantic Forest,” which is supported by a consortium of Brazilian research agencies, financed by CNPq PPBio Rede Bioma.

### Collection and morphological analyses of oocysts

Samples were collected during necropsy, directly from the large intestine. The fecal material was placed in a 15-ml conical centrifuge tube containing a 2.5% (*w*/*v*) solution of K_2_Cr_2_O_7_ and maintained at room temperature. On five consecutive days, the tube was opened and the solution was stirred vigorously to oxygenate it and promote the sporulation of any oocyst present in the faces. The samples were processed in a saturated sugar solution by the centrifugal flotation method (Sheather’s method), placed on slides examined under a microscope at a magnification of × 400 (Duszynski and Wilber 1997). Morphological observations and measurements were obtained using a Carl Zeiss Axio Scope.A1 binocular microscope with an apochromatic oil immersion objective lens and AxioVision imaging system. The oocysts were examined with an Imager.A2 light microscope Zeiss equipped with Nomarski interference contrast microscopy × 100 objective lenses, and the images captured with an AxioCam MRc. All measurements are given as the mean value in micrometers, followed by the range of values in parentheses.

### Histopathological analysis

To determine the site of infection, the small intestines of specimens positive for oocysts in their feces were segmented and fixed overnight in Carson Millonig formalin. The tissue samples were subsequently dehydrated in a progressive series of ethyl alcohol concentrations, diaphonized using xylol, and then embedded in paraffin blocks for the preparation of serial sections of 5 μm. The tissue sections were stained with hematoxylin and eosin and examined under a light microscope Carl Zeiss (Humason 1979). The developmental stages were photographed and measured, and then analyzed qualitatively and described based on the scheme of Kheysin (2013).

## Results

Four (7.5%) of the 53 *A*. *montensis* specimens analyzed were infected by an undescribed form of *Eimeria*. The histological analyses revealed the endogenous stages of the parasite in the small intestine of the mice, including the presence of macrogametocytes, microgametocytes, zygotes, and oocysts.

*Eimeria akodonensis* n. sp*.*

*Type host*: *Akodon montensis* Thomas, 1913 (Rodentia: Sigmodontinae), Symbiotype host (Frey et al. 1992), skin and skeleton, deposited in the National Museum of Rio de Janeiro (adult males, MNRJ nos. 83768, 83774, 83776, 83777).

*Type-locality*: Serra dos Órgãos National Park in Petrópolis, in the state of Rio de Janeiro, Brazil (22° 27′ 49″ S, 43° 05′ 14.09″ W).

*Type-material*: The oocysts were preserved in 70% ethanol, based on Duszynski and Gardner (1991). The samples were deposited in the Parasite Collection of the Department of Animal Parasitology (http://r1.ufrrj.br/lcc) at the Federal Rural University of Rio de Janeiro, in Seropédica, Rio de Janeiro, Brazil. Phototypes and line drawings were deposited together with the specimens. The catalog number is P-77/2017.

Sporulation time: Unknown.

*Site of infection*: Small intestine.

*Prevalence*: 4 of 53 (7.5%).

*Etymology*: The specific epithet is derived from the name of the host genus.\

Exogenous stage

**Fig. 1–3:**
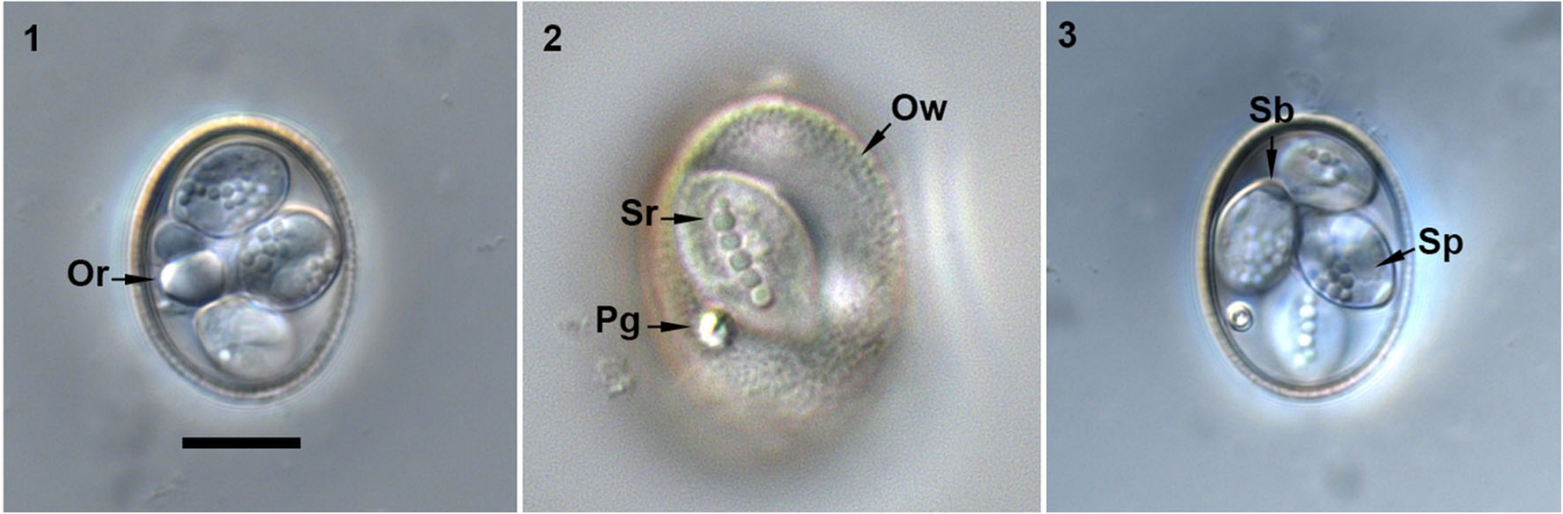
Nomarski interference-contrast photomicrographs of *Eimeria akodonensis* n. sp. from the montane grass mouse, *Akodon montensis*. Presence of oocyst residuum (Or) and highly refractile polar granule (Pg); Sporocyst residuum (Sr); wall (Ow); Stieda body (Sb); sporozoite (Sp). Scale bar = 10 μm

**Fig. 4:**
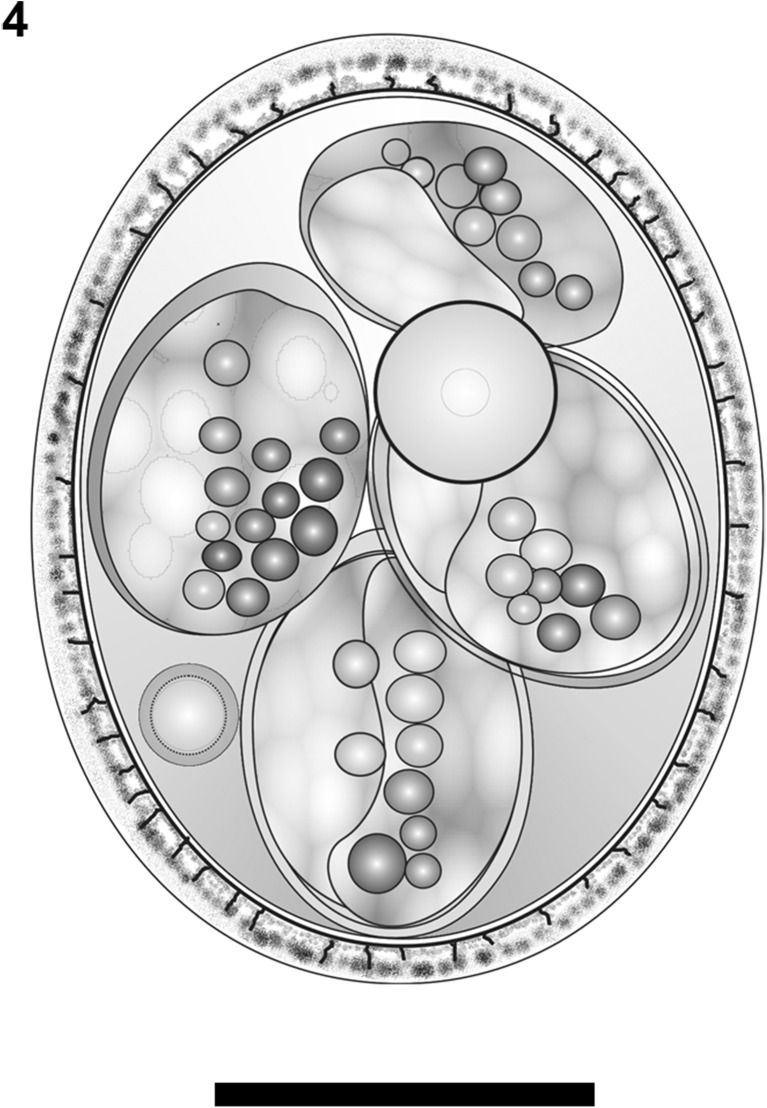
Composite line drawing of the sporulated oocyst of *Eimeria akodonensis* n. sp. Scale bar = 10 μm

Description (Figs. 1–3 and 4)

**Fig. 5–12:**
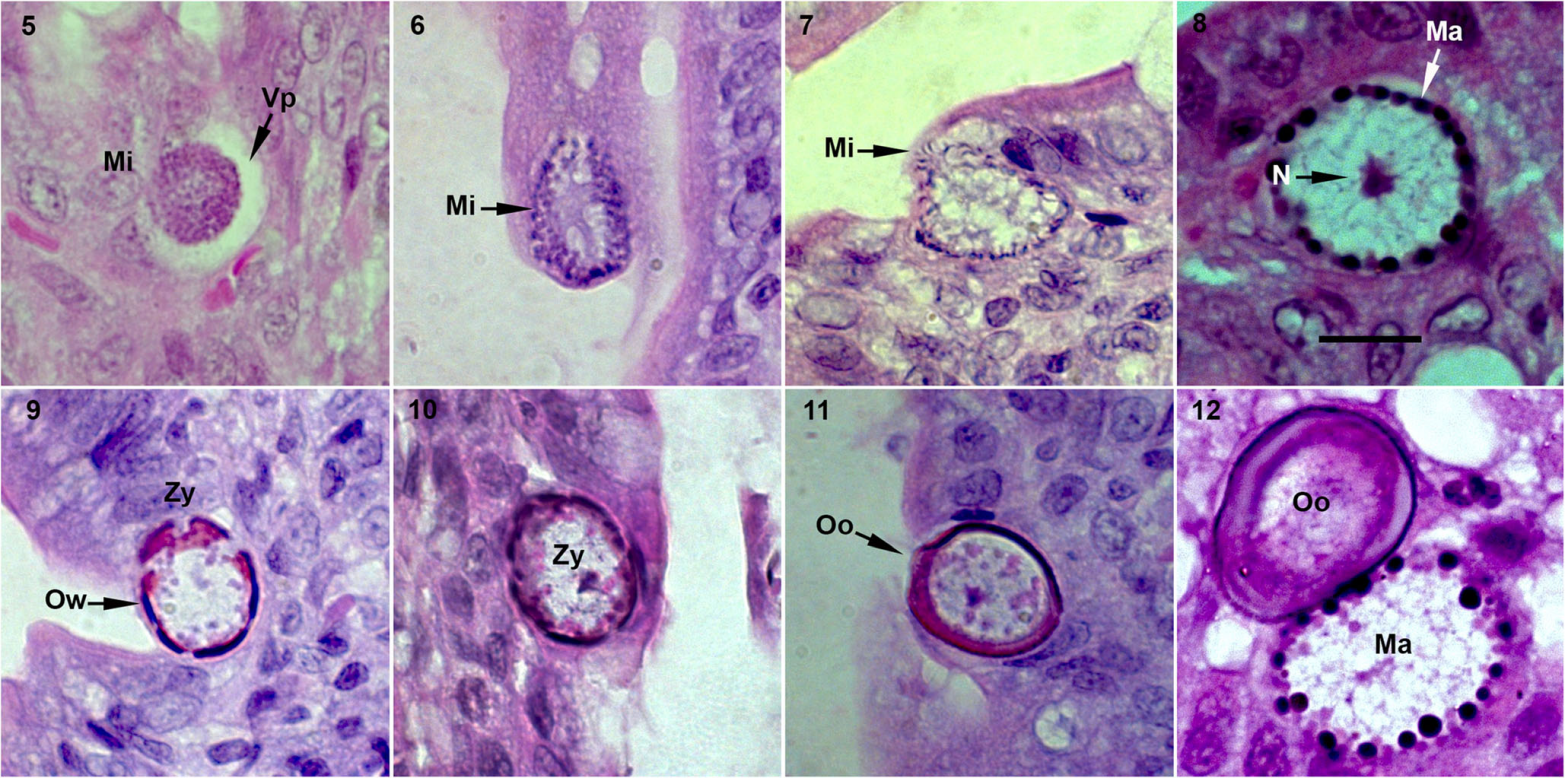
Light micrographs of the endogenous stages of *Eimeria akodonensis* n. sp. observed in the lamina propria of the small intestine of *Akodon montensis,* showing the microgamonts (Mi) and the parasitophorous vacuole in the host cell (Vp); microgametes (Mi); macrogamonts (Ma); nucleus (N) and the zygote (Zy) with wall-forming bodies arranged around its periphery (Ow); oocyst (Oo). Scale bar = 10 μm

Sporulated oocyst

Oocyst shape (*n* = 126) is ellipsoidal to subspherical, wall bi-layered, 1.5 μm (1.3–1.6) thick, outer layer rough. Oocyst length is 25.3 μm (21.0–28.0), with a width of 20.2 μm (17.0–22.0) and length/width (L/W) ratio of 1.3 μm (1.2–1.4). Polar granule is present, with oocyst residuum as a large spherical to subspherical globule.

Sporocyst

Sporocyst shape (*n* = 126)is ellipsoidal with length of 11.8 μm (9.3–14.4), width of 7.9 μm (6.7–9.3), and (L/W) ratio of 1.5 μm (1.4–1.7). Sporocysts with nipple-like Stieda body and sub-Stieda body are absent. A sporocyst residuum formed by several globules, usually along the sporocyst wall.

Endogenous stages

Description (Fig. 5–12)

The histological analysis revealed the endogenous development of the parasite in the jejunum portion of the small intestine. The infected cells of the lamina propria contained only parasites in the gametogenic phase, with the immature microgametocytes being enveloped by the parasitophorous vacuole, and having a rounded shape, approximately 10.9 μm (5.4–16.8) in length and 11.8 μm (5.5–17.3) in width (Fig. 5). Free immature micro-gametocytes and microgametes were also observed (Figs. 6–7), as were macrogametes at different stages of development, including the formation of the oocyst wall from the granules, approximately 20.9 μm (15.9–25.7) in length and 16.2 μm (12.6–19.9) in width, sub-spherical to ellipsoidal in shape (Figs.8–10). Mature and immature oocysts were also observed (Figs. 11–12).

## Discussion

In Brazil, the coccidian diversity found in wild rodents is still poorly known, since only three species were reported infecting rodents of the subfamily Sigmodontinae (Rodentia: Cricetidae): *Eimeria oryzomysi* from specimens of Oryzomyini rodents **(**Carini 1937), *Eimeria zygodontomyis* in *Necromys lasiurus* (Lainson and Shaw 1990), and *Besnoitia akodoni* in *Akodon montensis* (Dubey et al. 2003). In Venezuela, *Eimeria akodoni* was described in the grass mouse *Necromys urichi* J. A. Allen and Chapman, 1897 (Arcay-de-Peraza 1981), while *Eimeria ojastii* Arcay-de-Peraza, 1964 was recorded from *Nephelomys albigularis* Tomes, 1860 (Arcay-de-Peraza, 1964).

The morphology and morphometry of *Eimeria akodonensis* n. sp. were compared with data for coccidia of the family Eimeriidae described from other cricetid hosts in Brazil and Venezuela (Table 1). In the case of the study of Arcay-de-Peraza (1964), some data were missing, although they were not crucial to the comparison with *Eimeria akodonensis* n. sp.. *Eimeria oryzomysi* (Carini 1937) has the same shape as *Eimeria akodonensis* n. sp., but is slightly smaller, on average (22–25 μm × 17–19 μm vs. 21–28 μm × 17–22 μm), and does not have a polar granule, its wall is smooth and not very thick, and the residua of the sporocysts are made up of fine granules. *Eimeria ojastii* (Arcay-de-Peraza, 1964) is considerably smaller (17.4 × 13.2 vs. 25.3 × 20.2), ellipsoidal in shape, and its sporocyst does not have a Stieda body; no data were presented on the presence or absence of a polar granule. *Eimeria zygodontomyis* (Lainson and Shaw 1990) is also much smaller (16.5 μm × 12 μm vs. 25.3 μm × 20.2 μm), significantly different in shape, and have a fine, single-layered wall (0.6 vs. 1.5 μm) in comparison with the rough double wall of *Eimeria akodonensis* n. sp.

**Table 1:** Comparative morphology of *Eimeria akodonensis* n. sp. and other *Eimeria* species recorded from rodents from Brazil and Venezuela. The metrical data are given in micrometers as the mean followed by the range in parentheses. When no data were provided, or the data are inadequate for comparisons, the variable is shown as NR = Not Reported

Host	Species	Reference, country	Oocyst shape	Oocyst size L × W	Oocyst wall thickness	Oocyst shape index	Polar granule	Sporocyst size L × W	Stieda body/sub-Stieda body
*Akodon montensis*	*Eimeria akodonensis* n. sp*.*	Present study, Brazil	Ellipsoidal to subspherical	25.3 × 20.2 (21.0–28.0 × 17.0–22.0)	Bi-layered 1.5 (1.3–1.6)	1.3 (1.2–1.4)	Present	11.8 × 7.9 (9.3–14.4 × 6.7–9.3)	Present /absent
*Euryoryzomys* sp.	*E*. *oryzomysi*	Carini (1937), Brazil	Ellipsoidal to subspherical	NR (22–25 × 17–19)	Bi-layered NR	1.25	Absent	11 × 8 NR	Present /absent
*Nephelomys albigularis*	*E*. *ojastii*	Arcay-de-Peraza (1964), Venezuela	Ellipsoidal	17.4 × 13.2 NR	Bi-layered NR	NR	NR	7.5 × 5.9 NR	Absent/absent
*Necromys urichi*	*E*. *akodoni*	Arcay-de-Peraza (1981), Venezuela	Fusiform to ellipsoidal	27 × 18 NR	Tri-layered (1.5)	NR	Present	14 × 7 NR	Present /absent
*Necromys lasiurus*	*E*. *zygodontomyis*	Lainson and Shaw (1990), Brazil	Ellipsoidal to cylindrical	16.5 × 12 (13.7–18.7 × 11.2–12.5)	Uni-layered (0.6)	1.4 (1.2–1.5)	Present	8.4 × 5.5 (7.4–8.7 × 5.0–6.2)	Present /absent

Morphologically, the most similar of the four species to *Eimeria akodonensis* n. sp. is *E*. *akodoni*, which was described by Arcay-de-Peraza (1981) parasitizing *N*. *urichi*. The two species are similar in size (27 μm × 18 μm vs. 25.3 μm × 20.2 μm) and both have a polar granule, although the sporocysts are slightly different in their morphology and size (14 μm × 7 μm vs. 11.8 μm × 7.9 μm). Arcay-de-Peraza (1981) describes the oocysts of *E*. *akodoni* as fusiform, and when they were observed in immersion, their thick wall was apparent along their whole length except for the extremities, where they become thinner, taking on the appearance of “false opercules,” in the words of the author. This characteristic is not observed in *Eimeria akodonensis* n. sp., and the oocysts are not fusiform in shape. Arcay-de-Peraza describes the presence of three walls in *E*. *akodoni* and the absence of residuum in the oocyst, whereas in *Eimeria akodonensis* n. sp., the oocyst has a double wall, with a large residuum. Despite these broad morphological similarities, the endogenous development of *Eimeria akodonensis* n. sp. occurs in different portions of the intestine in comparison with *E*. *akodoni*. Whereas *Eimeria akodonensis* n. sp. develops in the small intestine, Arcay-de-Peraza (1981) found *E*. *akodoni* in the large intestine. According to Arcay-de-Peraza (1981), merogony and gametogony of *E*. *akodoni* occurred in epithelial cells of the large intestine. In the case of *E*. *akodonensis* n. sp., gametogony was only recorded in the jejunum portion of the small intestine. The microgametocytes of *E*. *akodonensis* n. sp. were smaller (10.9 × 11.8 μm) than *E*. *akodoni* (30.6 × 20.4 μm). In addition, macrogametocytes of *E*. *akodonensis* n. sp. were sub-spherical, 20.9 × 16.2 μm (L/W ratio: 1.2 μm), and those of *E*. *akodoni* were ellipsoidal, 19.7 × 11.7 μm (L/W ratio: 1.6 μm). The effects of infection by *Eimeria akodonensis* n. sp. on the intestinal tissue of *Akodon montensis* varied considerably, from a total absence to a light and diffuse infiltration distributed in the intestinal mucosa composed of neutrophils and mononuclear cells. However, this infiltration was not found in association with the parasites themselves, but rather in the epithelium of the intestinal villi. Overall, the set of morphological and physiological characteristics found in *Eimeria akodonensis* n. sp. supports conclusively its description as a new species.
